# Novel fluorescent nano-sensor based on amino-functionalization of Eu^3+^:SrSnO_3_ for copper ion detection in food and real drink water samples

**DOI:** 10.1039/d1ra01190a

**Published:** 2021-05-22

**Authors:** Z. Ghubish, R. Kamal, Hala R. Mahmoud, M. Saif, H. Hafez, M. El-Kemary

**Affiliations:** Institute of Nanoscience & Nanotechnology, KafrelSheikh University Kafr ElSheikh 33516 Egypt elkemary@nano.kfs.edu.eg elkemary@yahoo.com; Department of Chemistry, Faculty of Education, Ain Shams University Roxy Cairo 11711 Egypt; Natural Resources Department, Environmental Studies and Research Institute, University of Sadat City Egypt

## Abstract

Lanthanide-doped nanoparticles exhibit unique optical properties and have been widely utilized for different sensing applications. Herein, the Eu^3+^:SrSnO_3_@APTS nanosensor was synthesized and its optical properties were analyzed using UV-Vis and photoluminescence spectroscopy. The TEM images of the synthesized nanophosphor Eu^3+^:SrSnO_3_@APTS exhibited peanut-like morphology, composed of two or more spherical nanoparticles with an average diameter ∼33 nm. Effects of environmental pH values and doping concentrations as well as amino functionalization on the structure of Eu^3+^:SrSnO_3_ were investigated. The as-synthesized optical nanosensor was used for determination of copper ions based on a fluorescence quenching approach. Red emission with a long lifetime was obtained in the case of the 0.06 mol Eu^3+^:SrSnO_3_@APTS sample. Under the optimal experimental conditions, a Stern–Volmer plot exhibited a good linearity for copper ions over the concentration (0.00–10.8) × 10^−11^ mol L^−1^ with a correlation efficient of 0.996 and a limit of detection 3.4 × 10^−12^ mol L^−1^. The fluorescent sensor was dynamically quenched *via* a coulombic interaction mechanism between the Eu^3+^ (^5^L_6_) and Cu^2+^. The Eu^3+^:SrSnO_3_@APTS nanosensor with the optimal Eu^3+^ dopant concentration of 0.06 mol was applied for copper determination in food and real drink water samples with high recovery values. We believe that the developed nanosensor probe can also be used for the detection of other toxic compounds, with high selectivity and sensitivity.

## Abstract 2

Lanthanide-doped nanoparticles exhibit unique optical properties and have been widely utilized for different sensing applications.

## Graphical abstract

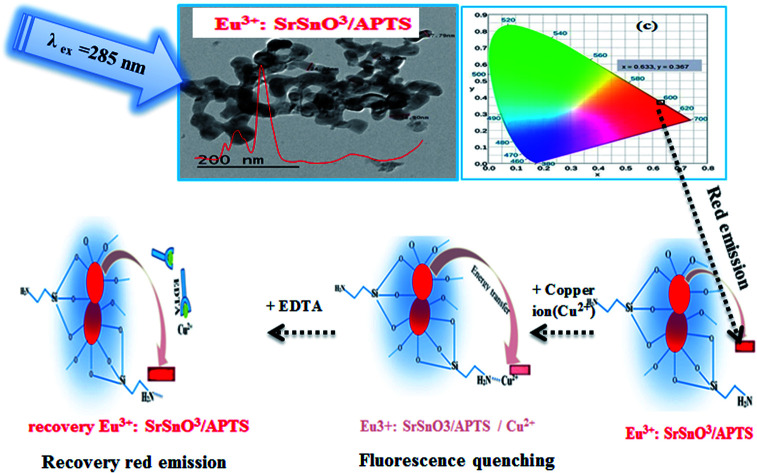

## 1. Introduction

Recently, the detection of copper ions in environmental and biological systems has attracted considerable attention because copper plays an important role in physiological processes of living organisms in certain doses.^1,2^ However, the overuse of copper is highly toxic and leads to several diseases such as kidney failure, nervous system damage, and several others.^3,4^ Moreover, at higher concentrations, Cu^2+^ can react with molecular oxygen to create reactive oxygen species (ROS), leading to significant damage to cell structures, due to its interaction with proteins, nucleic acids and lipids.^5,6^ Additionally, excess levels of copper in the body for two weeks or more may lead to adverse health effects as nausea, vomiting, diarrhea and permanent organs damage.^7^ Therefore, the detection of Cu^2+^ in food samples and drinking water is a necessary precaution. Consequently, production of sensitive and low-cost sensors for measuring copper in different industrial fields remains an active area of research.

Many analytical methods had been reported for the detection of copper ion. Among these methods are atomic absorption spectroscopy (AAS),^8^ inductively coupled plasma mass spectroscopy (ICP-MS), inductively coupled plasma atomic emission spectroscopy (ICP-AES),^9^ and electrochemical.^10,11^ Although these analytical methods are efficient quantitatively, they need expensive instrumentation and are time intensive. They also demand high operating cost, which makes these methods unsuitable for field monitoring.

In recent years, fluorescence methods had been widely employed for sensing various heavy metal ions,^12,13^ nanoparticles,^14^ fingerprint^15,16^ and biomarker,^17–19^ due to their low cost and visualization. Moreover, it has robust, high reliability, reusability, fast response time and other properties.^20^ Organic fluorescent sensors are limited by their photo-bleaching, broad emission band, short lifetime and low sensitivity.^21^

The simple, selective and sensitive nanopolar sensor based on a polyamine decorated β-cyclodextrin was used for the detection of copper(ii) in real environmental samples.^22^ However, inorganic fluorescent sensors attracted much attention, as it avoiding the above-mentioned disadvantages. It was reported that functionalized silver nanoparticles, CdSe coated with 4-mercaptobenzoic acid^23^ and ZnS quantum dots functionalize with l-cystine were used as a fluorescent sensor for copper detection. Although these inorganic sensors are simple, high-selective and relatively cheap, their broad emission bands showed some aggregation affecting the sensing ability. Moreover, the sensing features of silver and Q-dot depends on their shape and size, therefore the use of monodispersed nanoparticles is essential.^24^

Inorganic sensors based on lanthanide ions have been recently studied as an effective sensor for different applications. Lanthanide ions have distinct fluorescent properties that include anti-Stokes shifts, sharp emission peaks,^25^ and long-live luminescence.^26,27^ Lanthanide doped alkaline earth stannates MSnO_3_ (M = Sr, Ca, Ba) exhibit unique properties including lower chemical toxicity, lack of radioactive elements and greater thermal and chemical stability.^28^ Note that the forbidden f–f transition of the Ln(iii) ions, which induces significantly low molar extinction coefficient (*ε* < 10 M^−1^ cm^−1^) limiting the practical applications of the lanthanides.^29^ However, the presence of aromatic chromophores as linkers for lanthanides lead to minimize this effect and provide excellent sensing sensitivity toward the analyte.^29^ Due to their unique luminescence properties, lanthanide-based probes have recently used for the detection of nitroaromatic compounds in water,^30^ ions, trace-water, gas and temperature.^31^

In the present study, we have developed nano-fluorescent sensor based on SrSnO_3_:Eu^3+^@APTS as an efficient analytical tool with quick response, high selectivity and sensitivity for the detection of copper ion in food and drinking water samples.

## 2. Experimental

### 2.1 Materials

All chemicals used in the present study were of analytical grade and used without further purification. Metal salts: europium nitrate Eu(NO_3_)_3_ was prepared by reactions of europium oxide (Belami Fine Chemical) and nitric acid. Strontium chloride SrCl_2_, copper chloride CuCl_2_·2H_2_O, lead nitrate Pb(NO_3_)_2_, nickel nitrate hexahydrate Ni(NO_3_)_2_·6H_2_O, ferric chloride hexahydrate FeCl_3_·6H_2_O, magnesium nitrate Mg(NO_3_)_2_, manganese chloride MnCl_2_, mercuric nitrate Hg(NO_3_)_2_, tin(iv) chloride pentahydrate SnCl_4_·5H_2_O and cobalt chloride CoCl_2_ were obtained from Sigma. 3-Aminopropyl trimethoxysilane (APTS) as a silylating agent, purchased from Fluka. Acetic acid, NaOH and hydrochloric acid were obtained from Sigma-Aldrich.

### 2.2 Synthesis of fluorescent materials

#### 2.2.1 Synthesis of *x* mol Eu^3+^:SrSnO_3_

25 mL of aqueous solution of NaOH (1.2 mol L^−1^) was added to 25 mL of aqueous solution of SnCl_4_ (0.2 mol L^−1^) under stirring and a clear aqueous solution was obtained. The obtained solution was added drop wise into 50 mL of aqueous solution of SrCl_2_ (0.1 mol L^−1^), which contains different molar ratios (0.00–0.10 mol) from europium nitrate (Eu(NO_3_)_3_·6H_2_O). Then, 0.1 mol L^−1^ HCl was added with continued stirring for 30 min to adjust different pH. The obtained slurry was washed repeatedly with distilled water to remove excess salts. The product was finally dried at 80 °C in air and annealed at 700 °C.^32,33^

#### 2.2.2 Synthesis of SrSnO_3_:0.06 mol Eu^3+^@APTS

SrSnO_3_:Eu^3+^ (500 mg) was dispersed and sonicated in 600 mL ethanol/water (5/1) for 20 minutes. Acetic acid was added dropwise to the solution until the solution pH equal to 4. Then 4 mL of APTS was added to the solution and the mixture stirred for 3 hours at room temperature. Finally, the nanoparticle was purified and washed several times with ethanol and then dried for 24 hours at 45 °C in an drying oven. The obtained particles was characterized by FT-IR spectra.

### 2.3 Sensitivity of nanofluorescent sensor

Stock solutions of metal salts were synthesized in distilled water. All dilute solutions were prepared from standard stock aqueous solutions of concentration 0.1 M of their respective metal ions. An aqueous solution of metal salt (5 mL) has been mixed with 0.005 g of nanosensor and stirred for 30 min. Then the nanopowder was centrifuged. The photoluminescence spectra of solutions were recorded at *λ*_ex_ = 285 nm.

### 2.4 Analytical application in food and drinking water samples

A food sample including 2.0 g of green tea or 2.0 g of black tea or 5.2 of tomato sauce were put into porcelain crucible and burned with flame. Three powder samples were calcinated at 550 °C for 4 h, then 10 mL of concentrated nitric acid was added, followed by adding 5 mL of hydrogen peroxide 30% and the mixture was heated for drying. Finally, 25 mL water were added. Aliquots of these solutions was subjected to the analytical determination.^34^ The fluorescent nano-sensor was immediately mixed with diluted 5 mL of the digested solution. Fluorescence intensity of the solution was measured at 610 nm and the total copper concentration was determined from the Stern–Volmer plot.

Drinking water samples were taken from Kafr ElSheikh governorate, Egypt. All water samples were filtered using 0.45 μm member filters before testing. 1 mL was taken and diluted to 10 mL distilled water and mixed with the fluorescent sensor for copper ion determination.

Results obtained by developed fluorescent sensor were compared to data obtained by standard method using Inductive Coupled Plasma Atomic Emission Spectrometry (ICP-AES) (Optima 5300DV, PerkinElmer type).

### 2.5 Characterization of *x* mol Eu^3+^:SrSnO_3_ and 0.06 mol Eu^3+^:SrSnO_3_@APTS

Nanofluorescent sensor was characterized by high resolution Transmission Electron Microscope (TEM, JEM-2100, JEOL). The XRD patterns were recorded using Shimadzu X-ray diffractometer with CuKα_1_ radiation (*k* = 1.54056 Å). The accelerating voltage of 40 kV and an emission current of 30 mA were used. The FT-IR spectra were recorded using JASCO-FT-IR 6800 spectrometer. The photoluminescence emission spectra were recorded at room temperature with a spectrofluorometer shimaduz RF5301PC. The luminescent lifetime of the prepared compounds was recorded using a PerkinElmer LS 55 luminescence spectrometer (USA). Surface charge of nanoparticles were calculated by Brookhaven zeta potential/particle size analyser.

## 3. Results and discussion

### 3.1 Structure, surface and morphology

#### 3.1.1 XRD

**Fig. 1:**
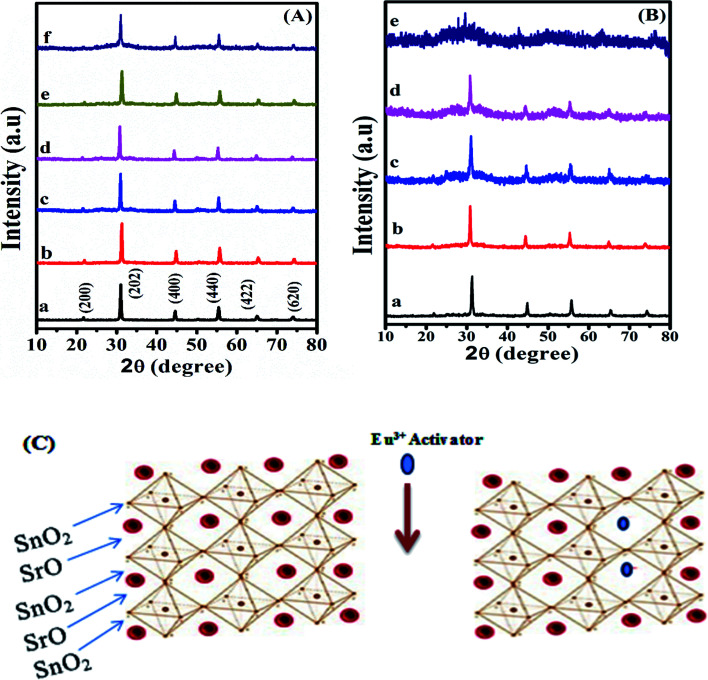
XRD spectra of (A) SrSnO_3_:*x* mol Eu^3+^ at pH = 12.5 and [*x*] of (a–f) = 0, 0.01; 0.02, 0.03; 0.06 and 0.10 mol, respectively and (B) SrSnO_3_:0.06 mol Eu^3+^ prepared at pH 12.5 (a), pH 11.0 (b), pH 10.5 (c), pH 10.5 + APTS (d) and pH 9 (e) and (C) schematic model of SrSnO_3_ and Eu^3+^:SrSnO_3_ cubic structure.

Fig. 1(A) showed the XRD patterns of the SrSnO_3_:*x* mol Eu^3+^ as a function of Eu^3+^ concentrations (*x* = 0.0–0.1 mol) (left) and pH values (right). The peaks observed at 21.5, 30.8, 44.4, 55.6, 64.9 and 73.8 can be related to (200), (202), (400), (440), (442) and (620) crystal planes of cubic phase (standard JCPDF card 09-0080). The lattice parameters of the prepared nanofluorescent samples were calculated^35^ and the obtained data are listed in Table 1. It is readily seen that, the host matrix exhibit a peak shift to the higher angles after addition of Eu^3+^ ion and no new peak was obtained, suggesting a small decrease in the unit cell parameters, Table 1. This phenomenon is likely a result of the shrinkage of the unit cell when Sr^2+^ ions were exchanged with smaller ionic radius Eu^3+^ ion (Sr^2+^ = 1.44 Å; Eu^3+^ = 1.12 Å),^36^Fig. 1(C). However, we have found that the crystal size of the nanophosphor decreases as a function of Eu^3+^ doping concentrations (pH = 12.5), Table 1.

**Table 1:** The crystal parameters and band gap values (*E*_g_) of *x* mol Eu^3+^:SrSnO_3_ (pH = 12.5) as a function of doping concentrations

SrSnO_3_:*x* mol Eu^3+^	2*θ* (°)	Crystal size (nm)	FWHM	Lattice parameter (Å)	Band gap (eV)
0.00	30.87	30.58	0.2816	8.1846	4.10
0.01	30.90	28.98	0.2974	8.0970	3.99
0.02	30.91	28.85	0.2985	8.0887	3.92
0.03	30.99	28.04	0.3072	8.0581	3.74
0.06	31.29	27.86	0.3093	8.0123	3.68
0.10	30.95	26.01	0.3311	8.0041	3.57

Fig. 1(B) shows the effect of pH of the synthesis solution on the crystal structure of SrSnO_3_:0.06 mol Eu^3+^ system. It is apparent that the observed peak became weaker and broader by decreasing the pH values from 12.5 to 9.0 and by addition of APTS, indicating a decrease on the crystal sizes of the obtained samples by decreasing the pH values and by addition of APTS. It was also observed that the sample prepared at pH = 9.0 showed an amorphous structure. In addition, the observed peak shift to the lower angles reflects a small increase in the unit cell parameters, Table 2.

**Table 2:** The crystal parameters and band gap values (*E*_g_) of 0.06 mol Eu^3+^:SrSnO_3_ as a function of pH values and in the presence of APTS

pH	2*θ* (°)	Crystal size (nm)	FWHW	Lattice parameter (Å)	Band gap (eV)
pH = 12.5	31.29	27.86	0.3093	8.0123	3.68
pH = 11.0	30.89	27.76	0.3102	8.1390	3.59
pH = 10.5	30.78	27.66	0.3112	8.1860	3.28
pH = 10.5 + APTS	30.76	27.42	0.3141	8.2020	3.14
pH = 9.0	30.83	20.50	0.4200	8.1740	3.10

#### 3.1.2 Microstructures and morphologies analysis

**Fig. 2:**
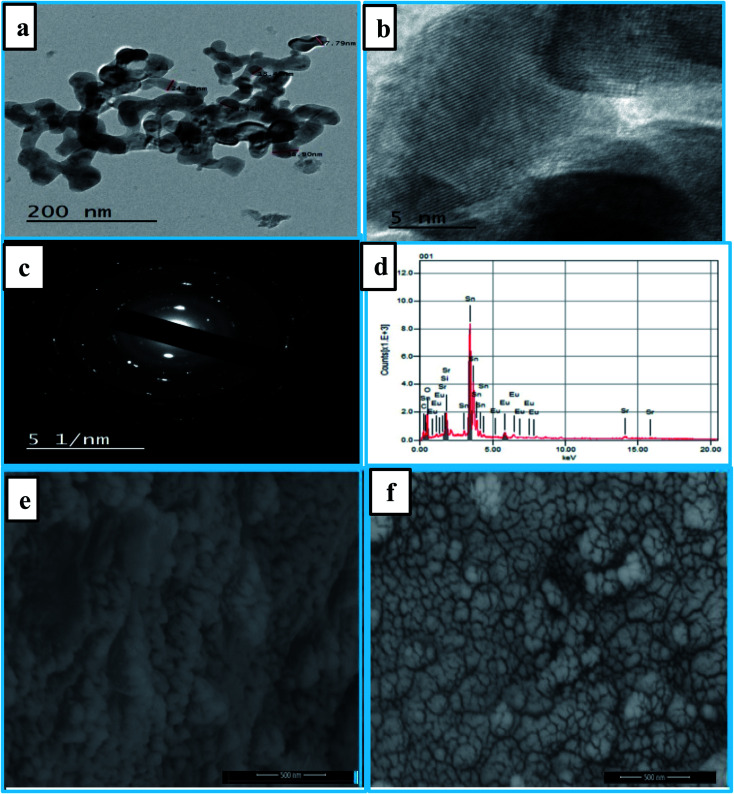
TEM images of the synthesized optimized nanophosphors Eu^3+^:SrSnO_3_@APTS (a and b). The SEM images of Eu^3+^:SrSnO_3_ (e), and 0.06 mol Eu^3+^:SrSnO_3_@APTS (f). The high resolution transmission electron microscopy (HR-TEM) of an interplanar *d*-spacing of lattice fringes (c); the energy dispersive X-ray (EDX) analysis of nanoparticles exhibited peaks corresponding to the presence of Eu, Sr, Sn, and O in addition to Si elements in case of amino-functionalized sample (d).

Fig. 2(a and b) shows transmission electron microscopy (TEM) images of the synthesized optimized nanophosphors Eu^3+^:SrSnO_3_@APTS. The particles exhibited peanut flower – like morphology, and each peanut flower like particle composed of two or more spherical nanoparticles with an average particle size ∼33 nm, which is smaller than that (∼45 nm) observed by C. Lee *et al.*^37^ The high resolution transmission electron microscopy (HR-TEM) showed the lattice fringes with an interplanar *d*-spacing of 0.28–0.31 nm, Fig. 2(c).

**Fig. 3:**
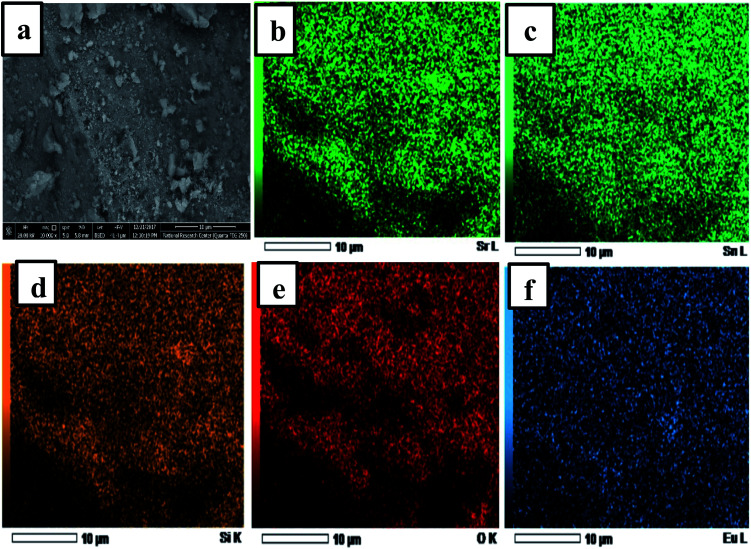
SEM image of 0.06 mol Eu^3+^:SrSnO_3_@APTS (a); EDS element mapping mono color of 0.06 mol Eu^3+^:SrSnO_3_:@APTS (all element), (b) Sr, (c) Sn, (d) Eu (e) O and (f) Si.

Fig. 2(e and f) displays the SEM images of samples Eu^3+^:SrSnO_3_ and 0.06 mol Eu^3+^:SrSnO_3_@APTS. The Eu^3+^:SrSnO_3_ exhibited an aggregated and irregular morphologies in micro domain, Fig. 2(e), while the amino functionalization sample (0.06 mol Eu^3+^:SrSnO_3_@APTS) has a uniform, regular peanut phase in nano-domain, Fig. 2(f). The energy dispersive X-ray (EDX) analysis of nanoparticles shows the corresponding peaks of Eu, Sr, Sn, and O in addition to Si elements Fig. 2(d). The elemental mapping demonstrating the homogeneous distribution of the elements in the investigated nano-phosphor samples, Fig. 3(a–f).

#### 3.1.3 Zeta potential

**Fig. 4:**
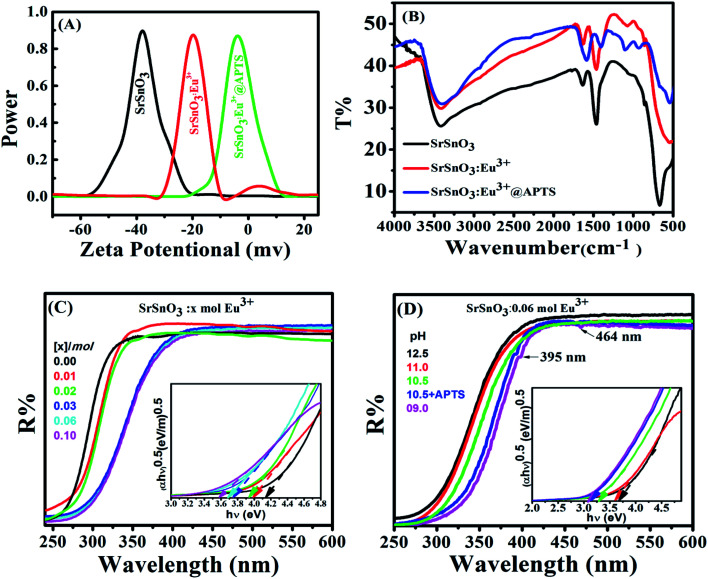
(A) Zeta potential measurements and (B) FTIR spectra of SrSnO_3_, 0.06 mol Eu^3+^:SrSnO_3_ and 0.06 mol Eu^3+^:SrSnO_3_@APTS samples prepared at pH = 10.5 in aqueous suspension. The diffuse reflectance UV/Vis spectra of (0–0.1) mol of Eu^3+^:SrSnO_3_ (C) and 0.06 mol Eu^3+^:SrSnO_3_ at different pH values (D). Inset shows plot of (*ahν*)^2^*versus* photon energy (*hν*).

Fig. 4(A) shows that the initial SrSnO_3_ particles exhibited zeta potential value of – 33 mV. After doping with Eu^3+^ ions, the zeta potential value decreased to −17 mV. This observation could be due to the ability of Eu^3+^ ions to substitute for Sr^2+^ in the host lattice upon lanthanide doping, which will make an unbalanced charge on the matrix surface. Consequently, it increases the surface polarity, positivity and chemical reactivity. However, the 0.06 mol Eu^3+^:SrSnO_3_ functionalized with APTS exhibited much lower zeta potential of about −3.0 mV, Fig. 4. This observation is probably, due to an abundance of APTS amine groups on the high positive 0.06 mol Eu^3+^:SrSnO_3_ surface.^38^ This leads to a high activated surface with low surface potential, which highlight the potential sensing application.

### 3.2 Optical properties

#### 3.2.1 FTIR

Fig. 4(B) shows the FT-IR spectra of samples SrSnO_3_, 0.06 mol Eu^3+^:SrSnO_3_ and 0.06 mol Eu^3+^:SrSnO_3_@APTS. SrSnO_3_ and 0.06 mol Eu^3+^. The SrSnO_3_ show broad peaks at 3400 cm^−1^ and 1454 cm^−1^ related to stretching and bending vibration of hydroxyl group –OH, respectively. The observed peaks at 853 and 517 cm^−1^ are associated to stannate group (SnO_3_^2−^) molecular vibrations.^39,40^

For 0.06 mol Eu^3+^:SrSnO_3_@APTS, the FTIR spectra revealed deformation mode of the Si–CH_2_ peak at 1415 cm^−1^ and the asymmetric stretching modes of the Si–O–Si bond at 1073 cm^−1^. Also, the scissoring absorption mode of the Si–O–Si siloxane groups exhibited a broad band at 570 cm^−1^. The observed peak at 1560 cm^−1^ indicates the presence of NH_2_ deformation modes of the amine groups, which are very strongly bound to the silanol groups *via* hydrogen bonded to form cyclic structures. The observed broad band at 3420 cm^−1^ is related to the stretching modes of NH_2_ and Si–OH groups. However, the weak peak at 2924 cm^−1^ is assigned to the stretching modes of CH_2_ group.^41^ These results clearly demonstrating the successful functionalization of in SrSnO_3_ with APTS.

#### 3.2.2 UV/Vis diffuse reflectance

Generally, the UV-Vis diffuse reflectance property depends on both doping concentration of Eu^3+^ and pH values during preparation procedures. Fig. 4(C and D) shows the UV-Vis diffuse reflectance spectra of Eu^3+^:SrSnO_3_ as a function of Eu^3+^ doping concentrations (0–0.1 mol) and pH values (12.5–9.0). The spectra of Eu^3+^:SrSnO_3_ exhibited an intense band in the UV region, which may be attributed to the transition of electrons located in the valence band of O 2p states into Sn 5s states of the conduction band upon absorption of UV light.^42^ This reflectance band exhibited red shift as a Eu^3+^ concentration increased. In addition, the SrSnO_3_ sample doped with 0.06 mol% Eu^3+^ also exhibit red shift as the pH value decreased. Moreover, a characteristic f–f transition bands of Eu^3+^ appeared in visible region of the spectra (395 nm, 419 nm and 533 nm) especially at low pH values.

The optical band gap (*E*_g_) of the samples can be calculated on the basis of the optical absorption spectra by using the following equation:

1 (*ahν*)^*n*^ = *β*(*hν* − *E*_g_)

where *hν* is the photon energy, *A* is the absorbance, *β* is related to the effective masses associated with the valence and conduction bands, and *n* is either equal to 2 for an indirect allowed transition or 1/2 for the direct allowed transition. The inset of Fig. 4(C and D) shows a good linear dependence of (*αhν*)^0.5^*versus* (*hν*) for all samples, suggesting that the samples have direct forbidden band gaps. The values of the band gap energy of the samples *n* were estimated using the linear extrapolation method and the estimated results are listed in Table 2.

It is readily seen that, the band gap value of SrSnO_3_ decreases with incorporation of Eu^3+^ ions and with decreasing the pH value during preparation procedures. However, the observed optical band gap value for optimized sample (0.06 mol Eu^3+^:SrSnO_3_@APTS) is 3.14 eV, which is relatively smaller than the reported value of bulk sample (4.3 eV).^12^ This behavior is due to a difference in particle size of the sample by different treatments. These observed UV-Vis diffuse reflectance data confirm the obtained results from XRD analysis.

#### 3.2.3 Photoluminescence study

**Fig. 5:**
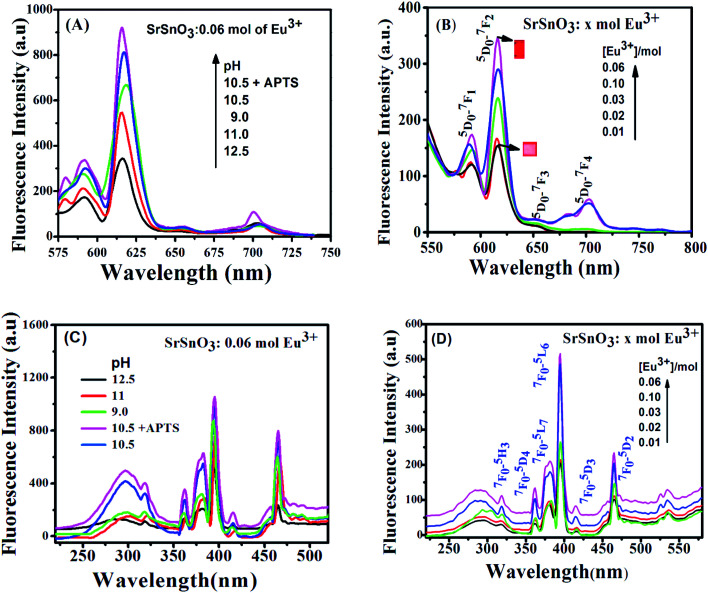
The excitation and emission spectra of (D) and (B) *x* mol Eu^3+^:SrSnO_3_ at different doping concentration (0.01–0.1 mole) prepared at pH 12.5; (C) and (A) 0.06 mol Eu^3+^:SrSnO_3_ at pH 12.5–9 and modified with APTS.

Fig. 5(B) displays the emission spectra (*λ*_ex_ = 285 nm) of Eu^3+^:SrSnO_3_ at different doping concentrations of Eu^3+^ in the range 0.01–0.1 mol and keeping the pH fixed at 12.5. It is apparent that an increase of Eu^3+^ concentration to 0.06 mol% leads to an enhancement of the photoluminescence (PL) intensity and then decreases after further increase in the concentration of Eu^3+^ ions, due to the concentration quenching. This observation suggesting that 0.06 mol Eu^3+^ doped SrSnO_3_ host gave the higher PL intensity. Therefore, the optimal Eu^3+^ dopant concentration is 0.06 mol%. A similar effect have also been performed for excitation spectra (*λ*_em_ = 615 nm) of Eu^3+^:SrSnO_3_ at different doping concentrations of Eu^3+^ as shown in Fig. 5(D). As can be seen from Fig. 5(A), the PL spectra exhibited four distinct peaks at about 593, 618, 656 and 710 nm. These observations are due to 4f transitions of Eu^3+^ from the excited ^5^D_0_ level to ground ^7^F_*J*_ levels (*J* = 0, 1, 2, 3 and 4), respectively, which are in close agreement to some reported literature values for Eu^3+^ doped SrGd_2_O_4_ phosphors.^43^

The pH dependence of the PL intensity of the 0.06 mol Eu^3+^:SrSnO_3_ are shown in Fig. 5(A). The results demonstrated that the PL intensity increases with decreasing pH value from 9 to 10.5, then begin to decrease upon more increase in the pH value to 12.5. An increase on the doping concentration and amorphous structure at relatively low pH value (9.0) increases the non-radiative deactivation process and consequently leading to decreasing the energy transfer probability. In addition, the sample 0.06 mol Eu^3+^:SrSnO_3_@APTS at pH 10.5 exhibited the relatively higher intense excitation band, due to increasing energy transfer probability, Fig. 5(C). This means that the presence of amino group of APTS on the Eu^3+^:SrSnO_3_ surface act as another sensitizer for Eu^3+^ in addition to the SrSnO_3_ host. However, the high aggregated micro-morphology of Eu^3+^:SrSnO_3_ prepared without APTS increases the non-radiative transitions compared to the sample prepared in the presence of APTS (SEM results). This behavior agree with the XRD data of the sample prepared by addition of APTS, which show a decrease on the crystal.

The excitation spectra of Eu^3+^:SrSnO_3_ nano-phosphor were also characterized with sharp weak lines located at 364, 383, 395, 465 and 533 nm. They are related to the intra-configurational 4f–4f transitions of Eu^3+^ ions doped in the host lattice. These peaks are assigned to ^7^F_0_–^5^D_4_, ^7^F_0_–^5^G_2_, ^7^F_0_–^5^L_6_, ^7^F_0_–^5^D_3_ and ^7^F_0_–^5^D_2_ transitions, respectively.^44^

**Fig. 6:**
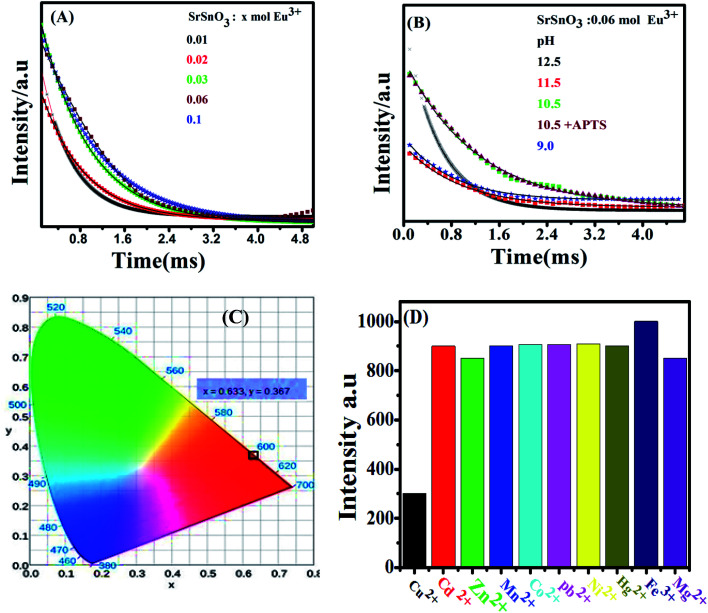
(A) The PL decay curves of (A) *x* mol Eu^3+^:SrSnO_3_ at different doping concentrations (0.01–0.1 mol) prepared at pH 12.5; (B) 0.06 mol Eu^3+^:SrSnO_3_ at pH 12.5–9, and (C) CIE diagram of 0.06 mol Eu^3+^:SrSnO_3_@APTS; (D) effect of different metal ions on intensity of Eu^3+^:SrSnO_3_@APTSA nanophosphor. Concentration of metal ions 10.8 × 10^−11^ M.

As shown in Fig. 6(B and D), the emission spectra obtained upon excitation of the phosphor at 285 nm are characterized with five emission bands at 580, 593, 616, 655 and 701 nm, which attributed to ^5^D_0_–^7^F_*J*_ (*J* = 0–4), respectively. However, the emission spectrum is broad and less-resolved, especially in case of 0.06 mol Eu^3+^:SrSnO_3_@APTS at pH 10.5. These observations are attributed to the nanostructure property of this sample, which changes the strength of local electrostatic field and site symmetry around lanthanide element due to structure disorder and surface defects.^45^ This suggestion is supported by increasing of red emission line relative to orange one and the asymmetry ratio values by decreasing the crystal size as a result of different modifications, Table 3.

**Table 3:** The measured PL and calculated Judd–Ofelt parameters of Eu^3+^:SrSnO_3_@APTS nano-phosphor as a function of a doping concentrations and pH values

Phosphors	Intensity		Asymmetric factor (*λ*_ex_ 285 nm)	*τ* (ms) ± error *λ*_ex_ = 285 nm	*A*_rad_	*A*_nrad_	Quantum efficient, %	*Ω*_2_ × 10^−20^	*Ω*_4_ × 10^−20^
	615 nm	590 nm							
***x* mol Eu** ^**3+**^									
0.01	153	118	1.30	0.550 ± 0.004	365	1453	20.0	1.93	0.14
0.02	167	125	1.34	0.890 ± 0.017	287	832	25.6	2.3	0.49
0.03	241	174	1.38	0.785 ± 0.003	352	999	26.1	2.5	0.68
0.06	338	173	1.97	1.030 ± 0.007	389	737	34.4	3.2	1.1
0.1	285	156	1.83	0.890 ± 0.009	338	773	30.0	3.3	1.4

**pH value**									
12.5	338	174	1.97	1.030 ± 0.007	389	737	34.4	3.2	1.8
11.0	558	214	2.60	0.925 ± 0.025	402	431	48.1	4.0	1.2
10.5	870	303	2.65	1.208 ± 0.024	488	498	50.7	4.4	1.3
10.5 + APTS	932	341	2.70	1.2 ± 0.008	414	418	50.2	3.9	1.9
9.0	850	307	2.41	0.802 ± 0.024	330	503	39.6	3.8	1.8

We note that high asymmetry value was obtained in case of 0.06 mol Eu^3+^:SrSnO_3_@APTS at pH 10.5. This is attributed to the altering of the coordination sphere of Eu^3+^ ions resulting from the further coordination of amino group of APTS to the Eu^3+^ ions on the surface.

Fig. 6(A and B) shows the photoluminescence decay curves of Eu^3+^:SrSnO_3_ as a function of doping concentrations and pH values upon excitation at 285 nm. Table 3 presents the obtained data of the monoexponential fits of europium ion (^5^D_0_ → ^7^F_2_). It is apparent that the PL lifetime of Eu^3+^ ions strongly depends on the doping concentration of Eu^3+^ ions. It first increases with increasing the doping concentration of Eu^3+^ ions and reaches its maximum value at around 0.06 moles and then decreases as a result of the concentration quenching effect. Furthermore, the PL lifetime values increase with decreasing pH value and reaches to the maximum value at pH = 10.5 and decrease at pH = 9. This means that SrSnO_3_:Eu^3+^@APTS at pH = 10.5 has a high energy transfer probability in which the amino group act as sensitizer in addition SrSnO_3_ host.^46,47^

**Scheme 1:**
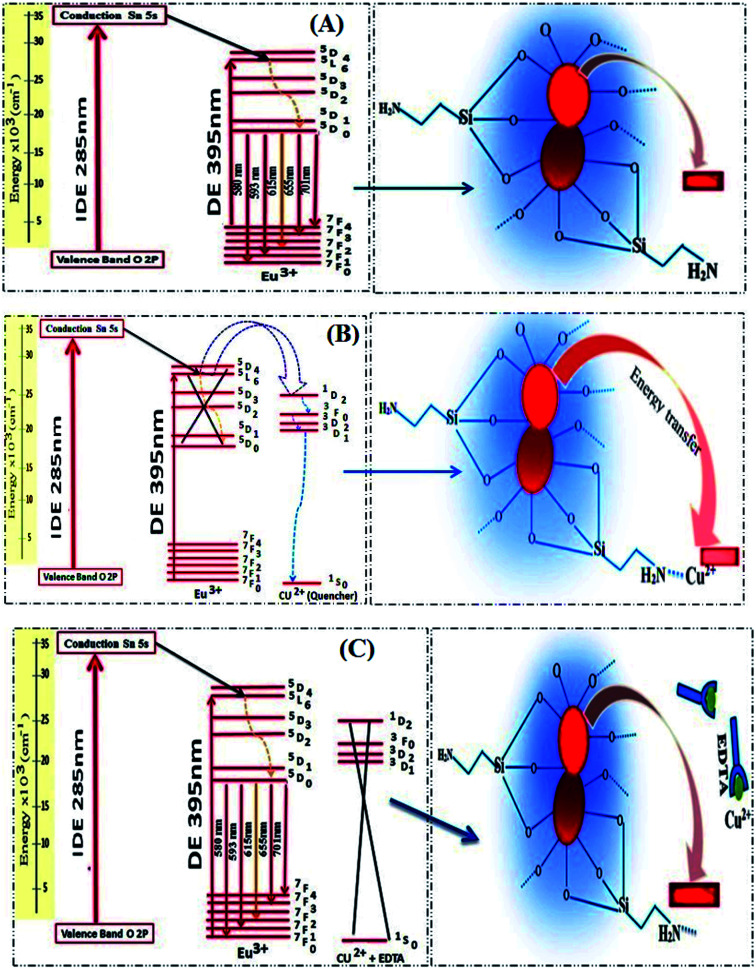
Proposed guest–host energy transfer mechanism in the in Eu^3+^:SrSnO_3_ nanophosphor (A) and quenching energy transfer process in the presence of Cu^2+^ ion (B) as well as after addition of EDTA (C).

The proposed energy transfer mechanism is shown in Scheme 1(A), where the Eu^3+^ can be sensitized by two bath ways. The first way is *via* host indirect excitation (IDE) (285 nm) and then the host transfers its energy to the Eu^3+^ emitting state to emit its characteristic emission color. The second one is *via* direct f–f excitation (DE) at 395 nm.^48^ The Commission international De I-Eclairage (CIE) coordinates of 0.06 mol Eu^3+^:SrSnO_3_@APTS was evaluated to be *x* = 0.633 and *y* = 0.367, which is found in pure red color region, Fig. 6(C).^49^

The local structure, environmental surrounding the Eu^3+^ ion inside host nanomaterial was investigated on the basies of Judd–Ofelt (J–O) theory. There parameters are then evaluated from emission spectra and lifetime and using intensity parameters *Ω*_*k*_ (*k* = 2, 4, 6) of Eu^3+^ ion inside host nanomaterial according to the modified method based on Kodaira *et al.* approach.^50^ Because the ^5^D_0_ → ^7^F_6_ was not identified in the emission spectrum (forbidden transition), the *Ω*_6_ intensity parameter could not be determined. However, the intensity parameters *Ω*_2_ and *Ω*_4_ were calculated using the following relation:2
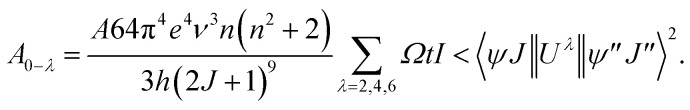
where *e* is the charge of an electron, *h* is Planck's constant (6.63 × 10^−27^ erg s), *ν* is the average transition energy (in cm^−1^), *n* is the refractive index of the medium and |〈^5^D_0_‖*U*^*k*^‖^7^F_*J*_〉|^2^ is the squared reduced matrix. Most of the matrix elements for transitions starting from the ^5^D_0_ level are zero.^51^ The ^5^D_0_–^7^F_2_ transition (*U*^(2)^ = 0.0028), the ^5^D_0_–^7^F_4_ transition (*U*^(4)^ = 0.002) and the ^5^D_0_ → ^7^F_6_ transition (*U*^(6)^ = 0.0002). The value of *U*_6_ is very small (negligible). *A*_0−*λ*_ can be calculated by using the following relation:3
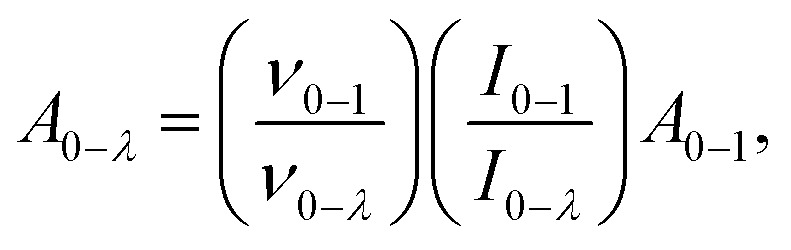


The sum of all *A*_0−*λ*_ provide the radiative decay rate (*A*_rad_), whereas inverse lifetime gives sum nonradiative and radiative decay rate (*A*_rad_/*A*_rad_ + *A*_nrad_). The parameters *Ω*_2_ and *Ω*_4_ are complex, and can be determined using a suitable calculation program such as JEOS and LUMPAC.^52,53^ The *Ω*_2_ values are related to the changing structure around Eu^3+^. For example, the very small value of *Ω*_2_ suggesting a greatly symmetric environment around the rare earth ion. However, *Ω*_4_ and *Ω*_6_ are relatively more sensitive to the variation in the macroscopic properties, such as viscosity and rigidity of the matrix.

The theoretically calculated *Ω*_2_ values are listed in Table 3. It should be noted that the theoretical values are in close agreement with the experimentally obtained values from PL spectra. The highest *Ω*_2_ values are obtained for 0.06 mol Eu^3+^:SrSnO_3_ prepared at pH = 10.5 as well as in the presence of APTS. This is due to the high distortion environment around surface Eu^3+^ cations polyhedra in the presence of SrSnO_3_ and APTS units.^54^ A similar effect has been observed for *Ω*_4_ parameter, which corresponds to the electron density on of the surrounding sensitizers, and its value increase with increasing the doping concentration and the highest value was observed for 0.06 mol Eu^3+^:SrSnO_3_@APTS at pH = 10.5, in the presence of APTS sensitizer, Table 3. The quantum efficiency values of nanophosphor increase with decreasing pH value and in the presence of APTS due to improved luminescence properties. These results are consistent with the obtained PL lifetime values.

### 3.3 Analytical performance and validation

Eu^3+^:SrSnO_3_ coated with APTS nanophosphor (Eu^3+^:SrSnO_3_@APTS) was tested as a fluorescent sensor for copper ion in aqueous solution. The coupling agent APTSA have been used because it change the surface charge of NPs, which may improve their stability in aqueous media. Therefore, Eu^3+^:SrSnO_3_@APTSA exhibited a characteristic intense pure red emission color with long lifetime value in aqueous medium (pH = 7) under UV illumination. We have studied the selectivity and sensitivity of Eu^3+^:SrSnO_3_@APTSA for different metal ions in aqueous medium (pH = 7). Fig. 6(D) shows the effect of different studied metal ions (10^−11^ M) on the fluorescence intensity of the Eu^3+^:SrSnO_3_@APTS nanophosphor. The red emission of Eu^3+^:SrSnO_3_@APTSA nanophosphor was highly quenched with 85% in the presence of copper ion compared to the other studied metal ions. This phenomenon may be due to the negative charge of the silanes.^55^ As a result, the amino group interacted with copper ion.

**Fig. 7:**
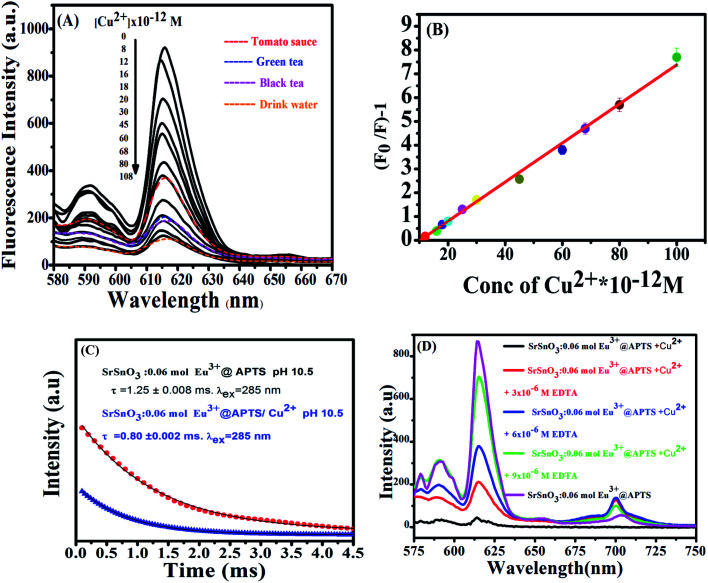
(A) Effect of different metal ions on PL intensity of Eu^3+^:SrSnO_3_@APTSA nanophosphor, [metal ions] = 10.8 × 10^−11^ M, (b) PL spectra of Eu^3+^:SrSnO_3_@APTS as a function of Cu^2+^ concentration (C) the Stern–Volmer plot of the quenching of Eu^3+^:SrSnO_3_@APTS nanophosphor by Cu^2+^ concentration; (D) the PL decay curves for 0.06 mol Eu^3+^:SrSnO_3_@APTS at pH = 10.5 in the presence and absence of Cu^2+^ ion.

Fig. 7(A) displays the change of emission spectra of Eu^3+^:SrSnO_3_@APTS nano-phosphor as a function of copper ion concentration in aqueous medium (pH = 7). As the Cu^2+^ ion concentration increases, the nanophosphor intensity decreases. The fluorescence quenching of nanophosphor was analyzed by the Stern–Volmer quenching plot using the following relation:^56,57^

4 *F*_0_/*F* = 1 + *K*_SV_[Q]

where, *F* and *F*_0_ are the fluorescence intensities of the nano-sensor in the absence and presence of quencher of concentration [Q], and *K*_SV_ is the Stern–Volmer quenching rate constant. Fig. 7(B) shows linear correlation between [(*F*_0_/*F*) − 1] and [Cu^2+^] plot in the concentration range 0.0–10.8 × 10^−11^ mol L^−1^ with *R*^2^ = 0.996. The slope of the straight line equal to *K*_SV_ = 8.216 × 10^10^ mol^−1^ L; standard deviation SD = 0.09523. The detection limit (LOD = from 3SD/*K*_SV_) and quantification detection limit (LOQ = 10SD/*K*_SV_) values of Cu^2+^ using the prepared nanomaterials was calculated to be equal to 3.4 × 10^−12^ mol L^−1^ and 1.16 × 10^−12^ mol L^−1^, respectively, which is lower than that of 1.3 mg L^−1^ reported for copper(ii) using atomic absorption spectrometry.^58^

The half quenching concentration (*C*_1/2_) was obtained from 1/*K*_SV_ = 1.217 × 10^−11^ mol L^−1^. The critical energy transfer distance (*R*_0_) between donor (Eu^3+^) and acceptor (Cu^2+^) was also calculated from the following relationship; *R*_0_ = 7.35/(*C*_1/2_)^1/3^ = 43 Å. Moreover, the luminescence decays of Eu^3+^:SrSnO_3_@APTS nanomaterials in the absence and presence of Cu^2+^ were measured after excitation at a wavelength of 285 nm for better understanding of quenching type. Lifetime values of Eu^3+^:SrSnO_3_@APTS before (*τ* = 1.25 ms) and after addition of Cu^2+^ (0.80 ms) was detected, Fig. 7(C). The luminescence lifetime and critical distance results demonstrated that the fluorescent sensor was dynamically quenched *via* coulombic interaction mechanism between the Eu^3+^ (^5^L_6_) and Cu^2+^ in which the Cu^2+^ excited state is well matched with an energy level ^5^L_6_ of Eu^3+^, Scheme 1(B). As a consequence, Cu^2+^ ions absorb the energy from the excited state of Eu^3+^. Furthermore, the complexation of –NH_2_ to Cu^2+^ ions decrease the distance between Cu^2+^ and Eu^3+^, which would significantly improve the efficiency of energy transfer between the ions.

As revealed before, the interaction of Cu^2+^ ions with NH_2_ of APTS coated on the surface of as-prepared NPs proceeds *via* energy transfer between NPs and Cu^2+^ ion. Support for this comes from adding EDTA as a complexing agent, and has stronger coordination ability to Cu^2+^ ions. As shown in Fig. 7(D), almost 85% of the initial PL intensity was recovered by adding different concentrations of EDTA. This result suggesting that the detection of Cu^2+^ ion by the as-prepared Eu^3+^:SrSnO_3_@APTS is reversible Scheme 1(C).

### 3.4 Interference with coexisting foreign substances

The anti-interference capacity of the new developed fluorescent nanosensor was evaluated to detect the performance of this new sensor. Under the optimum conditions, the influences of interference ions (Cl^−^, SO_4_^2−^, HCO_3_^−^, NO_3_^−^, C_6_H_5_O_7_^3−^, PO_4_^2−^, Cd^2+^, Ca^2+^, Mg^2+^, Fe^3+^, Co^2+^, Ni^2+^) on the fluorescence features were studied. As reported previously, any change in fluorescent intensity ≥ (±5%) the analytical signal value of Eu(iii) was considered as an interference.^34^ At fixing concentration of Cu(ii) at 10 ng L^−1^, the change in the analytical signal before and after addition of the interfering ion was determined and the calculated values for KCl, SO_4_^2−^, HCO^3−^, NO^3−^, citrate, PO_4_^2−^ did not interfere even at 1000 fold of Cu(ii); hence they were applied as a masking agent for Cd^2+^ at 10-fold levels, Ca^2+^, Mg^2+^, Fe^3+^ at 20-fold levels, Co^2+^ at 30 and Ni^2+^ at 35-fold, respectively. It is worth mentioning that the fluorescent nano-sensor exhibited high anti-interference of coexisting ions. The concentrations of these interfering ions were at least ten times higher than that of Cu^2+^ ion in the sample solution.

### 3.5 Application of SrSnO_3_:Eu^3+^@APTS in the determination of Cu(ii) in food samples

The optimized fluorescent nano-sensor (Eu^3+^:SrSnO_3_@APTS) was applied to copper sensing from food samples (tomato sauce, green and black tea) and wastewater samples were obtained from Kafr El Sheikh governorate, Egypt. These samples were measured in the absence and presence of Cu(ii), Fig. 7(A) and then analysed. The fluorescence spectra of nano-sensor were measured (*λ*_ex_ = 285 nm) after addition of all prepared food and drink water samples. The copper concentration was determined from the Stern–Volmer plot, Fig. 7(B), where three measurements were performed for each concentration and the results and the recoveries for samples are listed in Table 4. A good agreement was obtained between the added and found values of the analyte with high recovery values (average proposed/standard × 100). Moreover, the analyte samples (without any addition of external known Cu^2+^ concentration) were also analysed by ICP-OES for comparison and the results are summarised in Table 4. There results are in reasonable agreement and the *t*-test exhibited 95% confidence limit. The recovery results for the proposed method confirm the sensitivity of the nano-sensor for copper detection.

**Table 4:** Determination of Cu^2+^ in real samples

Sample	Cu^2+^ added (ng L^−1^)	Cu(ii) found by nano-sensor (ng L^−1^)	Standard (ICP-AES) (ng L^−1^)	Recovery (%)
Green tea	—	10.63	10.0	106.3
	1.36	12.12	—	102.0
	4.26	14.01	—	94.08
Black tea	—	10.44	10.0	104.4
Tomato sauce	—	5.70	6.00	95.00
	1.36	6.97	—	98.00
	4.26	10.03	—	104.0
Drink water	—	18.40	18.2	101.1
	1.36	18.95	—	96.8 0
	2.70	20.79	—	99.4 0

To further check the sensitivity of the proposed approach, we compared the observed results with the other values obtained other published methods as shown in Table 5. It is obvious that, our results are of lowest detection among the reported values. Thus, it may be concluded that the method is an effective approach detection of copper from various samples.

**Table 5:** Comparison of the characteristic data from literature

Sample	Method	Ions	LOD	Linearity	Ref.
Hydroxyapatite nanorods	FAAS	Cu^2+^	0.72 μg L^−1^	2.40–250 μg L^−1^	59
		Zn^2+^	0.55 μg L^−1^	1.83–300 μg L^−1^	
		Pb^2+^	5.12 μg L^−1^	17.06–400 μg L^−1^	
Hollow fiber-supported sol–gel combined with Dowex 50 W-x8	ICP-MS	Cu^2+^	1.1 μg L^−1^	0.1–140 μg L^−1^	60
		Zn^2+^	1.1 μg L^−1^	0.1–120 μg L^−1^	
		Pb^2+^	0.3 μg L^−1^	0.8–100 μg L^−1^	
Magnetic silica sorbent with polyelectrolyte multilayers on its surface	FAAS	Cu^2+^	0.23 μg L^−1^	1–30 μg L^−1^	61
Amberlite XAD-7 resin modified with CPDPINP	FAAS	Cu^2+^	1.6 μg L^−1^	10–180 μg L^−1^	62
		Zn^2+^	—	10–190 μg L^−1^	
		Pb^2+^	—	20–290 μg L^−1^	
SrSnO_3_:Eu^3+^@APTS	Luminescence method	Cu^2+^	3.4 × 10^−12^ M	(0.8–10) ×10^−11^ M	This work

## 4. Conclusions

New fluorescent nanosensor based on Eu^3+^:SrSnO_3_ was synthesized at different doping concentrations and pH values. The optimized nano-fluorescent sample was amino-functionalized with APTS to produce 0.06 mol Eu^3+^:SrSnO_3_@APTS, which has a characteristic surface, morphological and optical properties to be applied as nano-sensor material, which emits a pure red emission lines with long lifetime values. The results indicate that Eu^3+^:SrSnO_3_@APTS is useful nano-sensor for copper determination from different food and drink water samples with high sensitivity and selectivity.

## Conflicts of interest

The authors declared that they have no conflicts of interest to this work.

## Supplementary Material
